# GPs’ perspective on End-of-Life Care – an evaluation based on the German version of the General Practice End of Life Care Index

**DOI:** 10.3205/000286

**Published:** 2020-11-16

**Authors:** Katharina van Baal, Sophie Schrader, Birgitt Wiese, Siegfried Geyer, Stephanie Stiel, Nils Schneider, Gabriele Müller-Mundt, Kambiz Afshar

**Affiliations:** 1 Institute for General Practice, Hannover Medical School, Hannover, Germany; 2 Medical Sociology Unit, Hannover Medical School, Hannover, Germany

**Keywords:** general practice, primary care, palliative care, health services research, quality of health care

## Abstract

**Objective:** General practitioners (GPs) play a key role in the provision of general outpatient palliative care (AAPV) for the majority of patients at the end of life. The aim of this study was to evaluate the quality of End-of-Life Care (EoLC) from a GPs’ perspective using the German version of the General Practice End of Life Care Index (GP-EoLC-I).

**Methods:** Between autumn 2018 and spring 2019, all registered and eligible GPs in two counties in Lower Saxony (n=190) were asked to participate in a survey on EoLC using the German version of the self-assessment questionnaire GP-EoLC-I. The index comprises two subscales: *clinical care* (13 items) and *practice organisation* (12 items). The summated index of both subscales measures the quality of EoLC by GPs (25 items; range 14–40). The questionnaire was supplemented by questions on sociodemographic data, indicators for good palliative care (PC) and requirements to improve PC. Quantitative data were analysed by descriptive statistics and free text answers by conventional content analysis according to Hsieh and Shannon.

**Results:** 52 GPs (females: n=16) of 34 practices (single practices: n=26) participated in the study. The mean GP-EoLC-I was 27.5 (SD 4.5). The items revealed potential for improvement: systematic identification of patients with potential PC needs, multidisciplinary case conferences to discuss PC patients, application of care protocols and symptom assessment tools, documentation of patients’ wishes and beliefs as well as inclusion of family and carers. Regarding the indicators for good PC, the most relevant indicators from the GPs’ perspective were collaboration and coordination, integration of relatives, advance care planning and documentation. As requirements to improve PC, GPs highlighted further training and the use of standardised tools such as instruments to support the systematic identification of PC patients.

**Conclusions:** To our knowledge for the first time in Germany, an internationally tested self-assessment questionnaire measuring the quality of EoLC by GPs was applied. The GP-EoLC-I in this study was slightly lower than the index of GPs in the United Kingdom. Including relatives and family carers, implementing tools to support early identification of PC patients and strengthening cooperation between GPs and other stakeholders in PC may be promising approaches to improve general PC and EoLC in Germany.

## Zusammenfassung

**Ziel:** Hausärzt*innen (HÄ) spielen eine Schlüsselrolle in der allgemeinen ambulanten Palliativversorgung (AAPV). Ziel dieser Studie war es, die Qualität der Versorgung am Lebensende aus Sicht von HÄ mit Hilfe der deutschen Version des General Practice End of Life Care Index (GP-EoLC-I: „Hausärztliche Palliativversorgung und Begleitung am Lebensende“) zu erfassen.

**Methoden:** Zwischen Herbst 2018 und Frühjahr 2019 wurden alle in zwei Landkreisen in Niedersachsen registrierten und geeigneten HÄ (n=190) um Teilnahme an einer Selbsteinschätzung zur Versorgung am Lebensende mit Hilfe des GP-EoLC-I gebeten. Dessen Index umfasst zwei Subskalen: *Klinische Praxis* (13 Items) und *Praxisorganisation* (12 Items). Der Index wird durch Summation beider Subskalen gebildet und gilt als Maß für die Qualität der Versorgung am Lebensende durch HÄ (25 Items, Spannweite 14–40). Der Fragebogen wurde durch Fragen zu soziodemografischen Daten, zu Indikatoren für eine gute Palliativversorgung (PV) und Voraussetzungen für eine Verbesserung der PV ergänzt. Quantitative Daten wurden anhand deskriptiver Statistiken und Freitextantworten mittels konventioneller Inhaltsanalyse nach Hsieh und Shannon ausgewertet.

**Ergebnisse:** 52 HÄ (weiblich: n=16) aus 34 Praxen (Einzelpraxen: n=26) nahmen an der Studie teil. Der mittlere GP-EoLC-I lag bei 27,5 (SD 4,5). Die Items zeigten Verbesserungspotenziale auf: systematische Identifikation von Patient*innen mit potentiellem palliativem Bedarf, multidisziplinäre Fallbesprechungen zur Diskussion von Patient*innen mit palliativem Bedarf, Anwendung von Handlungsempfehlungen und Erfassungsbögen zur Symptomlast, Dokumentation von Wünschen und Vorstellungen der Patient*innen sowie die Integration von Angehörigen. Bezüglich der Indikatoren für eine gute PV waren die Zusammenarbeit und Abstimmung, die Integration von Angehörigen sowie frühzeitige Versorgungsplanung und Dokumentation aus Sicht der HÄ besonders relevant. Als Voraussetzungen für eine Verbesserung der PV betonten die HÄ eine weitergehende Ausbildung und den Gebrauch von standardisierten Tools wie beispielsweise Instrumenten zur systematischen Identifikation von Palliativpatient*innen.

**Schlussfolgerungen:** Unseres Wissens nach kam erstmalig in Deutschland ein international erprobter Fragebogen zur Selbsteinschätzung der Qualität der hausärztlichen Versorgung am Lebensende zum Einsatz. Der GP-EoLC-I war in dieser Studie etwas geringer als der Index der HÄ im Vereinigten Königreich. Die Einbindung von Angehörigen, die Implementierung von Instrumenten zur frühzeitigen Identifikation von Patient*innen mit potenziellem palliativem Bedarf und die Stärkung der Zusammenarbeit zwischen HÄ und anderen an der PV beteiligten Akteur*innen könnten vielversprechende Ansätze für eine Verbesserung der allgemeinen ambulanten PV und der Versorgung am Lebensende in Deutschland sein.

## Introduction

According to the World Health Organization, palliative care (PC) can be defined as *“an approach that improves the quality of life of patients and their families facing the problems associated with life-threatening illness, through the prevention and relief of suffering by means of early identification and impeccable assessment and treatment of pain and other problems, physical, psychosocial and spiritual*” [1]. The majority of people at the end of life can be treated within general palliative care (PC), while approximately 10–15% are in need of specialist PC [2], [3].

In Germany, general outpatient PC is most frequently provided by general practitioners (GPs) for patients with low or medium symptom intensity, while specialist outpatient palliative care (SAPV) is typically provided by interdisciplinary specialist palliative care teams for patients with particularly complex symptoms [4], [5], [6]. While specialist PC has developed substantially in the past decades in Germany, there are still no clear standards for the provision of general PC, especially for patients with non-malignant life-limiting chronic diseases such as chronic heart failure or chronic obstructive pulmonary disease [7], [8], [9]. Mitchell et al. described four main topics that are relevant for the provision of End-of-Life Care (EoLC) by GPs:

1. continuity of care including time, staff and workload issues;
2. patient and family factors with challenges containing early identification of PC needs, the possibility for care planning discussions and support for families;
3. medical management with symptom control issues and access to specialist services; and
4. expertise and training including development in knowledge, skills and attitudes concerning EoLC [10].

Major challenges identified by healthcare providers in general practice relate especially to three dimensions: *knowledge*, *professional attitude* and *skills* [11], [12]. Furthermore, a need for collaborative care with an inclusion of existing structures in general practices has been shown [11]. Consequently, assessments need to address educational and organisational elements, such as communication and collaboration with patients, caregivers and other stakeholders in PC.

It is well known that GPs play a key role in general PC [13]. The majority of GPs consider themselves to be essential actors in EoLC [10], [14]. The advantages of EoLC provided by GPs are amongst others defined by their good accessibility and by their significant role in home-based PC [15], [16].

Literature shows developments in measuring quality of PC by quantitative quality indicators, but further modification of these indicators is required [17]. In contrast to the United Kingdom (UK), existing data concerning the quality of EoLC provided by GPs in Germany is insufficient [18]. To date, no tool has been systematically applied in Germany to evaluate EoLC by GPs. The General Practice End of Life Care Index (GP-EoLC-I) is a standardised self-assessment instrument that allows for measuring the quality of EoLC by GPs [18].

An evaluation of practice organisation and clinical care in providing PC by GPs in Germany is the prerequisite for further studies directed towards the improvement of EoLC in primary care. Thus, the main objective of the present study was an evaluation of the quality of PC and EoLC in general practice from the GPs’ perspective. The following questions were to be answered in particular:

- How do GPs evaluate their practice organisation and clinical practice in providing EoLC?
- What are relevant indicators for a good PC in primary care from the GPs’ point of view?
- Which requirements do GPs consider relevant for a further improvement of PC in Germany?

## Methods

### Study design

This cross-sectional study is part of the research project “Optimal care at the end of life” (OPAL) [19], which aims to improve care at the end of life in a selected region in Lower Saxony, Germany.

### Setting and study population

The target group of this study were all GPs practicing in two counties of a rural region in Lower Saxony according to the registry of physicians. GPs that exclusively treated patients with a private health insurance were excluded. In October 2018, all eligible GPs in the target region were informed about the project and invited to take part in two steps: 1. by letter and 2. via phone. The requests via phone were maintained until every general practice accepted or refused their participation. If required, which was the case several times, additional information on OPAL was faxed to the general practices. The study material was brief, clear and consistent, including an OPAL logo for recognition purposes. These efforts were supplemented by presentations of the study on physicians’ conferences in the targeted region. The recruitment phase ended in April 2019.

### Survey

The two questionnaires assessed in this study are described in the following.

#### GP-EoLC-I

The GP-EoLC-I was developed in the UK by a multidisciplinary team at the University of Sheffield as part of a standardised questionnaire in a national survey to evaluate the implementation of key indicators of PC and EoLC. The questionnaire was designed in accordance with the recommendations of the National Institute for Clinical Excellence and the Gold Standards Framework (GSF) for primary care [18].

The GP-EoLC-I allows the evaluation of practice organisation and clinical care as well as the quality of PC and EoLC from the GPs’ point of view. The subscales *practice organisation* (12 items) and *clinical care* (13 items) are summed up to an index score (25 items). It can be considered as a measure for the quality of PC and EoLC [18]. For the calculation of the GP-EoLC-I, items with a four-staged scale are dichotomised according to the index calculation procedure developed for the original version by Hughes et al. [18]. Consequently, the subscale *practice organisation* can reach an index between 1 and 14 while the index for the subscale *clinical care* can range between 13 and 26. The overall GP-EoLC-I varies between 14 and 40. The higher the index, the better the quality of care by GPs can be considered. The original version of the GP-EoLC-I indicated a satisfactory level of internal reliability and internal consistency [18].

The GP-EoLC-I covers the so-called “7 Cs” defined in the GSF as central levels of action in PC [20]. These are:

1. communication,
2. co-ordination,
3. control of symptoms,
4. continuity of care,
5. continued learning,
6. carer support, and
7. care in the dying phase.

These aspects are relevant and feasible for general PC in Germany as well, and are partially equivalent to important quality indicators defined and reviewed by Engeser et al. [21].

The German version of the GP-EoLC-I “Hausärztliche Palliativversorgung und Begleitung am Lebensende” was systematically developed, tested and adjusted for the German context by the Institute for General Practice at Hannover Medical School [22] in agreement with the originators (copyright ^©^University of Sheffield 2010 funded by Macmillan Cancer Support; all rights reserved). The German version of the instrument comprises 34 items, thereof 25 items from the GP-EoLC-I. The nine additional items contained further questions on the GPs’ personal background, basic characteristics of the general practices, and a global question on the overall self-assessed quality of PC (response scale ranging from 1 “very bad” to 5 “very good”). Additionally, open-ended questions concerning relevant criteria for good PC and requirements for an improvement of PC from the GPs’ point of view were integrated.

#### Structural questionnaire

Apart from the GP-EoLC-I, a semi-structured questionnaire was applied to assess structural characteristics and further information on the patients treated in the participating general practices. It was completed only once for each general practice. In group or joint practices, the practice teams decided who should complete the structural questionnaire.

### Ethics and data security

Written informed consent was obtained from every GP prior to every assessment. GPs’ data were pseudonymised with an individual code and registered in a code list. This code list was stored separately from the data sheets. Only the study team had access to this data and any other study material.

### Data analysis

The data was analysed using the Statistical Package for Social Sciences Version 26 (SPSS Inc., Chicago, IL/USA). Descriptive statistics of quantitative data included the calculation of median and interquartile range (IQR), mean and standard deviation (SD), and percentages.

Open-ended questions were analysed by conventional content analysis as described by Hsieh and Shannon [23]. The categories developed in this process were reviewed by two authors.

Missing items were not replaced. In these cases, a decreased sample size was stated for that particular item. There were no missing data in the 25 items building the GP-EoLC-I.

## Results

### Recruitment of the participants

**Figure 1:**
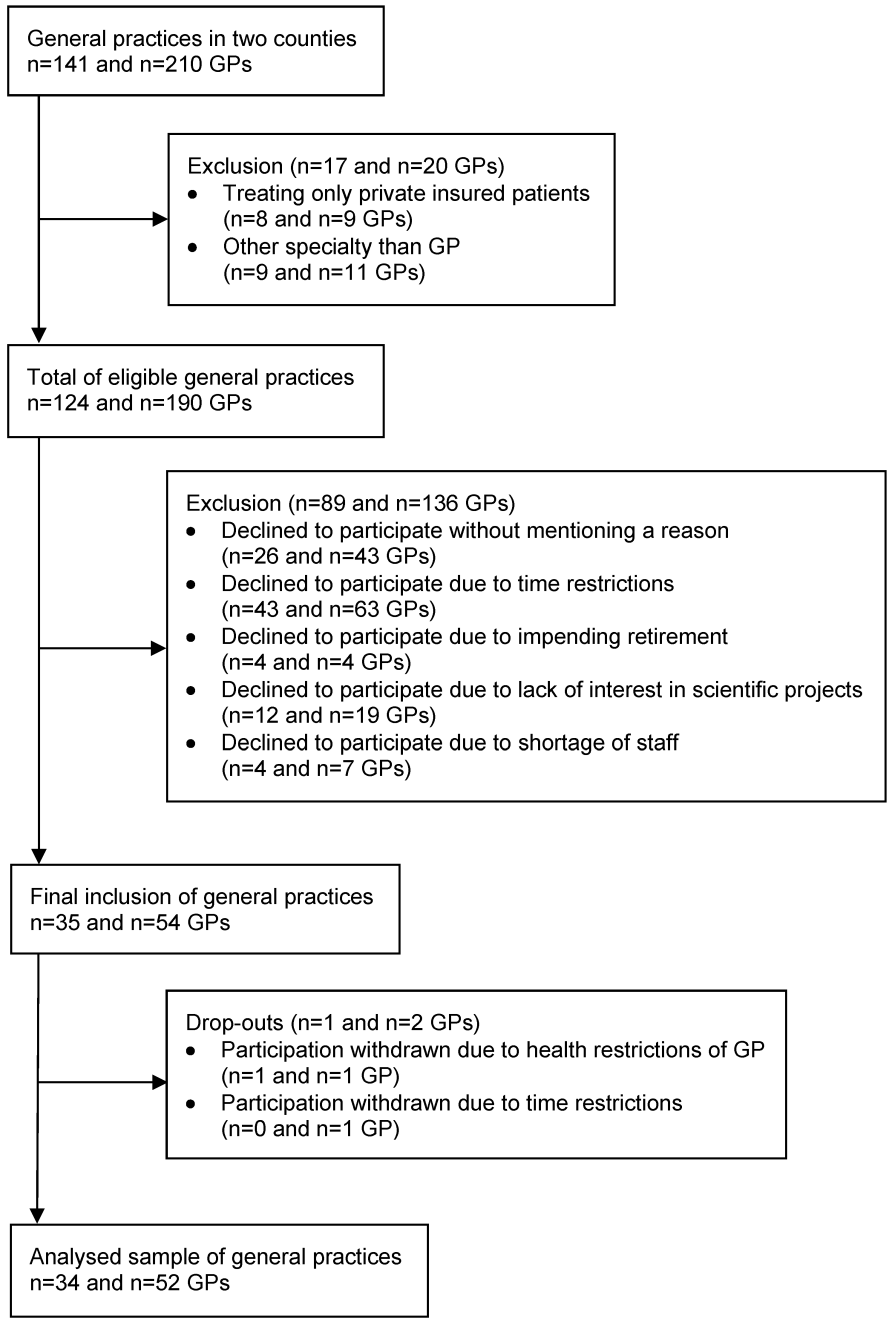
Flow chart for the recruitment of GPs and general practices

In autumn 2018, 190 GPs from 124 eligible general practices were contacted (contacts per practice: median 4; IQR 2–5). A total of 52 GPs from 34 general practices took part in the study (recruitment rate: 27.4% of all eligible general practices). Figure 1 (Fig. 1) outlines the recruitment of GPs. Data collection was completed in June 2019.

### Description of the study sample

The 34 participating general practices were located in a rural region in Lower Saxony (Hameln-Pyrmont/Schaumburg). The study sample consisted of 36 male (69.2%) and 16 female (30.8%) GPs aged between 29 and 77 years. The number of GPs in general practices varied between one (n=20, 58.8%) and eight (n=1, 2.9%). Further information about the study sample is shown in Table 1 (Tab. 1) and Table 2 (Tab. 2). Table 3 (Tab. 3) presents further characteristics of the 34 general practices, especially on the patient population treated in these practices.

### GP-EoLC-I

In the given sample, the overall GP-EoLC-I showed a median value of 28.0 (IQR 25.0–31.0; mean 27.5, SD 4.5).

Table 4 (Tab. 4) shows the results of the descriptive analysis of the items for the subscale *practice organisation*. A median value of 7.0 (IQR 5.0–8.0; mean 6.8, SD 2.1) was revealed for the whole sample.

Most GPs did not or only sometimes systematically identify patients with palliative needs in the case file (69.2%). The presence of a malignant diagnosis (78.8%) was not the only criterion relevant for the initiation of PC. GPs stated that they also took life-limiting non-malignant diseases (73.1%) and terminal diseases (96.2%) into account when initiating PC. A minority of GPs discussed PC patients in formal regular meetings (4 GPs, 7.7%), occasional meetings (3 GPs, 5.8%) or informal regular discussions (6 GPs, 11.5%). Most GPs discussed PC patients in an ad hoc liaison (32 GPs, 61.5%). After all, 26.9% of GPs in this sample did not discuss PC patients in one of these multidisciplinary forums at all. Most GPs did not have a system for coordination of PC (65.4%), a named coordinator for PC (86.5%) or a unified regional record for PC patients (69.2%). Also, the use of a protocol for the care of dying cancer patients (67.3%) and of tools for an assessment of PC needs (88.5%) was mostly missing. However, the majority of GPs had a system to make anticipatory medication available out-of-hours (76.9%).

Table 5 (Tab. 5) shows the results of the items for the subscale *clinical care*. The median value of the subscale *clinical care* was 21.0 (IQR 19.0–23.0; mean 20.8, SD 3.3). The majority of the GPs in this sample always or mostly recorded care plans for patients with PC needs (67.3%), encouraged patients with PC in preparing for death in a self-determined manner (69.2%), and assisted them in addressing unfinished business (75%) as well as in preparing advance directives (75%). The majority of GPs always or mostly routinely assessed and discontinued inappropriate interventions including medication (92.3%), and recorded a named family carer for discussion and coordination of care (82.7%). A slight majority of GPs always or mostly provided a handover form for out-of-hours care with information about PC patients (57.7%). Around two-thirds of GPs (63.4%) was available to PC patients out-of-hours in the final phase. In contrast, a minority of GPs provided appropriate written information for family and carers (21.1%). A slight majority of GPs sometimes or rarely/never recorded the wishes or spiritual beliefs concerning the dying process (57.7%), recorded the preferred place of care at the end of life/place of death (59.7%) or routinely documented impending death (59.6%). Having a documentation of the family/carers’ insights into the patients’ condition was confirmed by 25 GPs indicating doing it always or mostly (48.1%). 27 GPs did this sometimes or rarely/never (51.9%).

### Self-estimated quality of PC

While seven GPs (13.5%) estimated the quality of their own PC as very good and 24 GPs (46.2%) as good, 17 (32.7%) considered the quality of PC as satisfactory. Four GPs did not evaluate themselves.

### Indicators for a good PC

A total of 46 GPs stated at least one indicator for a good PC. Especially time-related factors such as the accessibility and availability of GPs and other institutions in PC (e.g. SAPV teams, nursing staff and hospice staff) were considered as relevant factors. GPs rated advance care planning and an early identification of PC needs as important but also time-consuming in daily practice routine. The early planning and the recognition of a need for action was also a timely matter reported by the GPs (indicator “planning and documentation”). Furthermore, GPs highlighted the relevance of good collaboration and coordination for adequate PC. This included collaboration between the different PC institutions and other professionals as well as the integration of the patient and his or her relatives/carers. Humanity and empathy in patient-doctor communication were assessed to be exceedingly relevant. GPs also emphasised the relevance of symptom control, avoidance of hospitalisation and further training. After all, positive feedback from patients, their relatives/carers and other health care professionals were named as meaningful indicators for good PC. Categories including their quantity and exemplary quotations are listed in Table 6 (Tab. 6).

### Requirements for improvement of PC

In total, 41 of the 52 GPs who participated in the survey named at least one requirement for the improvement of PC. Financial incentives and educational factors as well as personnel- and time-related resources were considered to play a highly important role. GPs also highlighted the relevance of standardised concepts, such as a consequent and standardised documentation. After all, GPs deemed a change in awareness in society and within health care professionals necessary for an improvement of PC. Table 7 (Tab. 7) shows categories, their quantity, and examples for the mentioned requirements.

## Discussion

This study aimed to evaluate the quality of PC and EoLC in general practice in a rural region in Lower Saxony from a GPs’ perspective using the German version of the self-assessment questionnaire GP-EoLC-I.

The challenges based on the results of the GP-EoLC-I, the mentioned indicators for a good PC and requirements for an improvement of PC match most dimensions covered by the seven Cs in the GSF [20].

A potential for improvement was revealed within the dimensions *communication* and *continued* *learning* (e.g. lack of multidisciplinary practice forums and systematic identification of patients with PC needs). Additionally, *co-ordination* (e.g. lack of an assigned coordinator, coordination system and interdisciplinary collaboration and coordination) and *control of symptoms* (e.g. missing use of care protocols and symptom assessment tools, planning and documentation with standardised tools) showed potential for improvement. With regard to the subscale *clinical care* of the GP-EoLC-I, *care in the dying phase* (e.g. documentation of impending death, planning for the last phase of life) as well as *carer support* (e.g. appropriate written information for family and carers and integration of family and carers on all levels of care) revealed further potential for improvement.

### Comparison of the quality of PC and EoLC

This sample of German GPs showed a lower GP-EoLC-I with a difference of 3.5 points compared to the UK data from Hughes et al. (UK: GP-EoLC-I mean 31.0; SD 8.1) [18]. When comparing these two studies, several differences of the two health care systems in the UK and in Germany have to be considered. There is, for example, no formal gate-keeping for GPs in Germany, and no standards such as the GSF have been implemented nationwide.

However, comparing data from Germany with international data is needed for benchmarking and further development of the GP-EoLC-I. The median value assessed in this study equals 70% of the maximal index. This might be considered as moderate and matches the GPs’ estimation of their own usual PC and EoLC, where more than three-quarters of the GPs rated the overall quality of their PC and EoLC as satisfactory or good.

### Inclusion of family and carers

It is well known that family carers contribute substantially to the care of patients with terminal illness [24], [25]. Informal provision of care by family and other carers may be crucial for providing general PC in a domestic environment in the first place [26]. A complex set of social and emotional factors is involved in providing care at the end of life in home caregiving, and there are deficits in the support and service provision for family carers [27], [28]. In a previous study on GPs’ experiences in EoLC in the UK, the support of families and carers and specifically the provision of necessary information about an impending crisis was highly important [10].

Future approaches should include family and carers on all levels of care to promote an improvement of PC and EoLC in general, and to address support for family caregivers.

### Planning and documentation

The provision of PC differs for patients with malignant and non-malignant diseases; especially for patients with non-malignant diseases, several barriers for the initiation and provision of PC were described [7], [29], [30]. According to Murtagh et al., decisions about when PC should be provided are difficult, particularly for patients with volatile disease processes such as those with non-malignant diseases [30]. The initiation of PC was not limited to patients with cancer diagnoses in this sample, as life-limiting non-malignant diseases and terminal diseases were considered as important criteria as well. However, systematic identification of PC patients, as well as the utilisation of protocols and symptom assessment tools were missing in PC by GPs according to this study. These aspects might be promising approaches aiming at an improvement of PC in future research. Advance care planning and the use of standardised tools for the identification of patients with potential PC needs seem to be of high importance, which is also underlined by several other studies [31], [32]. Internationally, different tools are used to identify patients with PC needs, e.g. the Supportive and Palliative Care Indicators Tool (SPICT™) [33]. SPICT supports the identification of patients with deteriorating health. It has been translated and adjusted for the German context [34]. If systematically implemented, SPICT might be a practical and helpful tool for GPs in Germany.

### Cooperation and coordination

It has already been acknowledged that a formation of networks and the cooperation between GPs and other physicians or health professionals play a key role in general PC in Germany and internationally [26], [35]. A close collaboration and cooperation between GPs, SAPV teams and services, as well as community nursing services was the highest-ranked action to improve PC in Germany, which is why a strengthening of cooperation should be of high priority [36]. A good cooperation can positively influence patients’ outcomes, i.e. avoiding unplanned hospitalisation and futile treatment at the end of life [35].

However, determining the appropriate time to initiate PC remains a challenge for many physicians [37], [38]. Especially patients with non-malignant diseases often receive PC at late stages in their disease trajectory [7], [39]. Being aware of potential PC situations is a crucial step for the identification of patients who might benefit from PC and for the initiation of concrete PC actions [35], [40]. These aspects emphasise the importance of multidisciplinary forums and/or case conferences for the discussion of patients with PC needs, which seem to be missing according to the majority of GPs in our study.

Future interventional studies should aim at an improvement of cooperation and coordination between GPs and other health care professionals or institutions in PC, which may start with a development of a system for coordination of PC in general practices and with regular multidisciplinary practice forums and/or case conferences.

### Strengths and limitations

A strength of this study is the sample size of 52 GPs in 34 general practices, which is a satisfactory sample because of the regional approach. Great efforts were made for recruitment, when all registered GPs in the targeted region were contacted numerous times until a positive or negative reply was obtained. Therefore, a relative high recruitment rate compared to other questionnaire studies among GPs was achieved with 27.4% of general practices in the targeted region [41]. However, our results cannot be generalised blindly. Our sample size results in small subgroups (Table 1 (Tab. 1) and Table 2 (Tab. 2)). Thus, subgroup analyses were not reasonable.

Additionally, the German version of the GP-EoLC-I was used with a regional approach for the first time. It can be considered as useful and expedient for an evaluation of PC and EoLC by GPs and should be taken into consideration for utilisation in future research.

However, limitations are the possible self-selection and recall bias. Participants may be more likely to have an interest in the topic of the project and their insights may differ from those who refused to participate [42]. According to the Medical Association of Lower Saxony, 12.6% of registered GPs have an additional qualification in PC in Lower Saxony. Nine GPs (17.3%) in this sample had an additional qualification in PC, which reveals a slightly higher education in PC in this sample. Furthermore, a potential selection bias has to be taken into account, as our sample comprises a high number of GPs working in teaching practices of the Institute for General Practice (Table 2 (Tab. 2)). Due to selection bias and the regional approach, the results of this study cannot be generalised unreservedly.

After all, data about quality of PC by GPs was self-reported, which is why it has to be interpreted with caution. However, Hughes et al. found accordance between the results of the questionnaire and external data [18].

## Conclusion

To our knowledge for the first time, an internationally tested self-assessment questionnaire was used to measure the quality of PC and EoLC provided by GPs in Germany. The survey results indicate potentials for improvement in primary care for patients with PC needs. There is a particular need for targeted interventions aiming at an inclusion of families and carers. Aside from that, an early initiation of PC and systematic identification of PC needs by use of standardised tools is clearly required. Also, future interventions should focus on strengthening the coordination and cooperation between GPs and other PC stakeholders.

The results of this paper can be used to compare the quality of PC by GPs in different regions and as a pre-post comparison for interventional studies aiming at an improvement of general PC in Germany.

## Abbreviations

- AAPV: general outpatient palliative care
- EoLC: End-of-Life Care
- GP: general practitioners
- GP-EoLC-I: General Practice End of Life Care Index
- GSF: Gold Standards Framework
- IQR: interquartile range
- OPAL: optimal care at the end of life
- PC: palliative care
- SAPV: specialist outpatient palliative care
- SD: standard deviation
- SPICT: Supportive and Palliative Care Indicators Tool

## Notes

### Ethical statement

Ethical approval for this study was obtained by the Ethics Committee of Hannover Medical School on 16 August 2018 (registration no. 8038_BO_K_2018).

### Informed consent

GPs gave written informed consent prior to participation and any procedure in this study.

### Authors’ contributions

KA, GMM and NSch had the original idea for the conception of the study OPAL. KA, GMM and NSch substantially contributed to the development of the study design and have given relevant intellectual input. KvB, SoS and KA made substantial contributions to recruitment, collection, analysis, and interpretation of data. SSt, SG and BW gave input relating to data analysis and the appraisal of results. KvB and KA wrote the manuscript. All authors read, improved, and approved the final manuscript.

### Funding

This study is part of the project OPAL, funded by the innovation funds of the Federal Joint Committee (Innovationsfonds des Gemeinsamen Bundesausschusses, grant no. 01VSF17028). The funding does not have any influence on the design of the study, the data collection, data analysis and interpretation of data or on writing this manuscript.

### Competing interests

The authors declare that they have no competing interests.

### Data management and sharing

Data collected during study application is available from the corresponding author on reasonable request.

### Acknowledgements

We would like to thank our cooperation partners: the coordinators of the Health Region Hameln-Pyrmont, the Lower Saxony representation of the German Association of General Practitioners, the Centre for Competence and Coordination of Hospice and Palliative Care in Lower Saxony, the Local Health Care Fund Lower Saxony (AOK-N), and of course the participating GPs and their practice teams. We also wish to thank Sonja Riedel-Schatte and Esther Stahlke, who contributed in the recruitment, data collection and data input. We further thank Fabian Tetzlaff for giving advice on data analysis.

## Figures and Tables

**Table 1:**
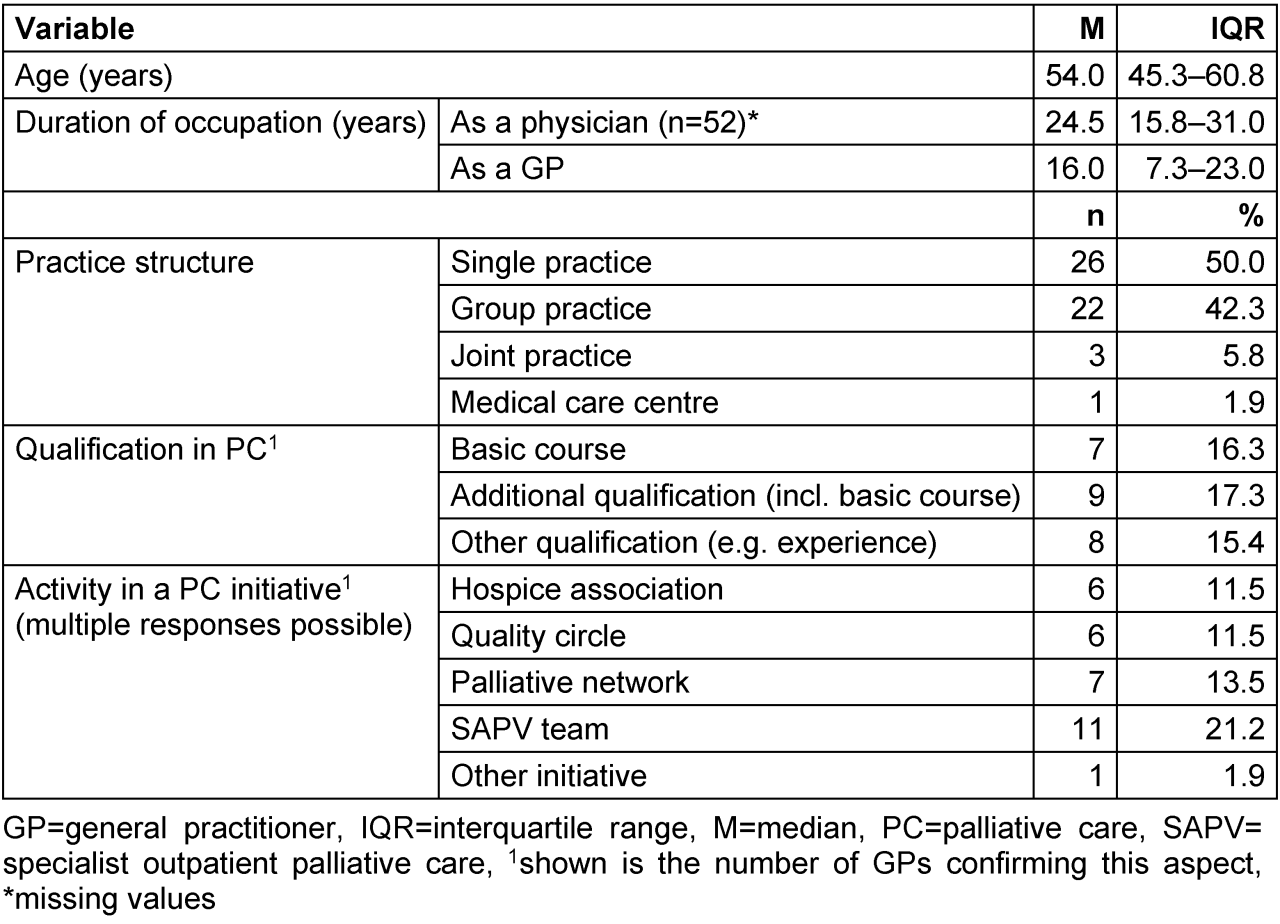
Description of the study sample on GP level (n=52)

**Table 2:**
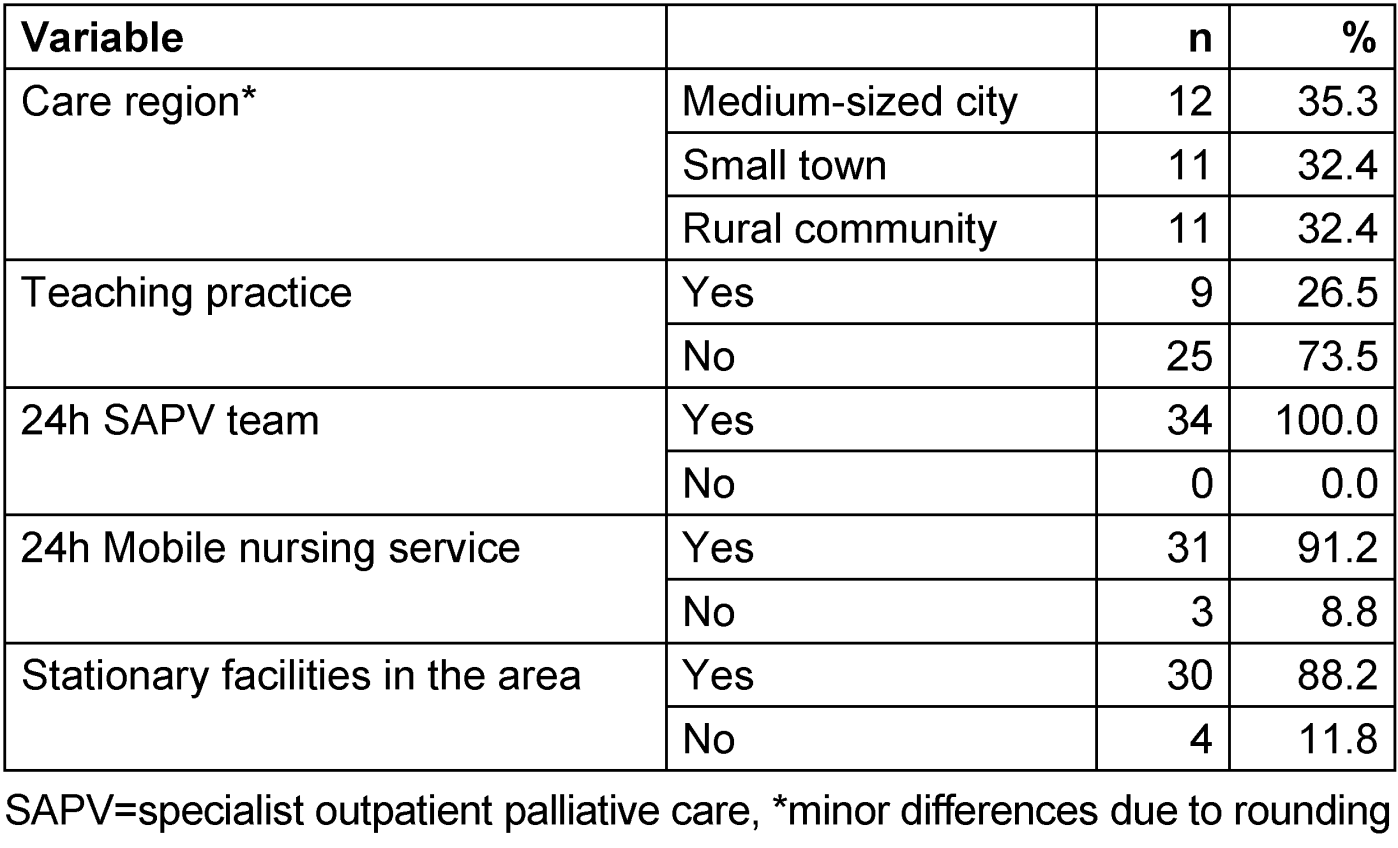
Description of the study sample on the level of the general practices (n=34)

**Table 3:**
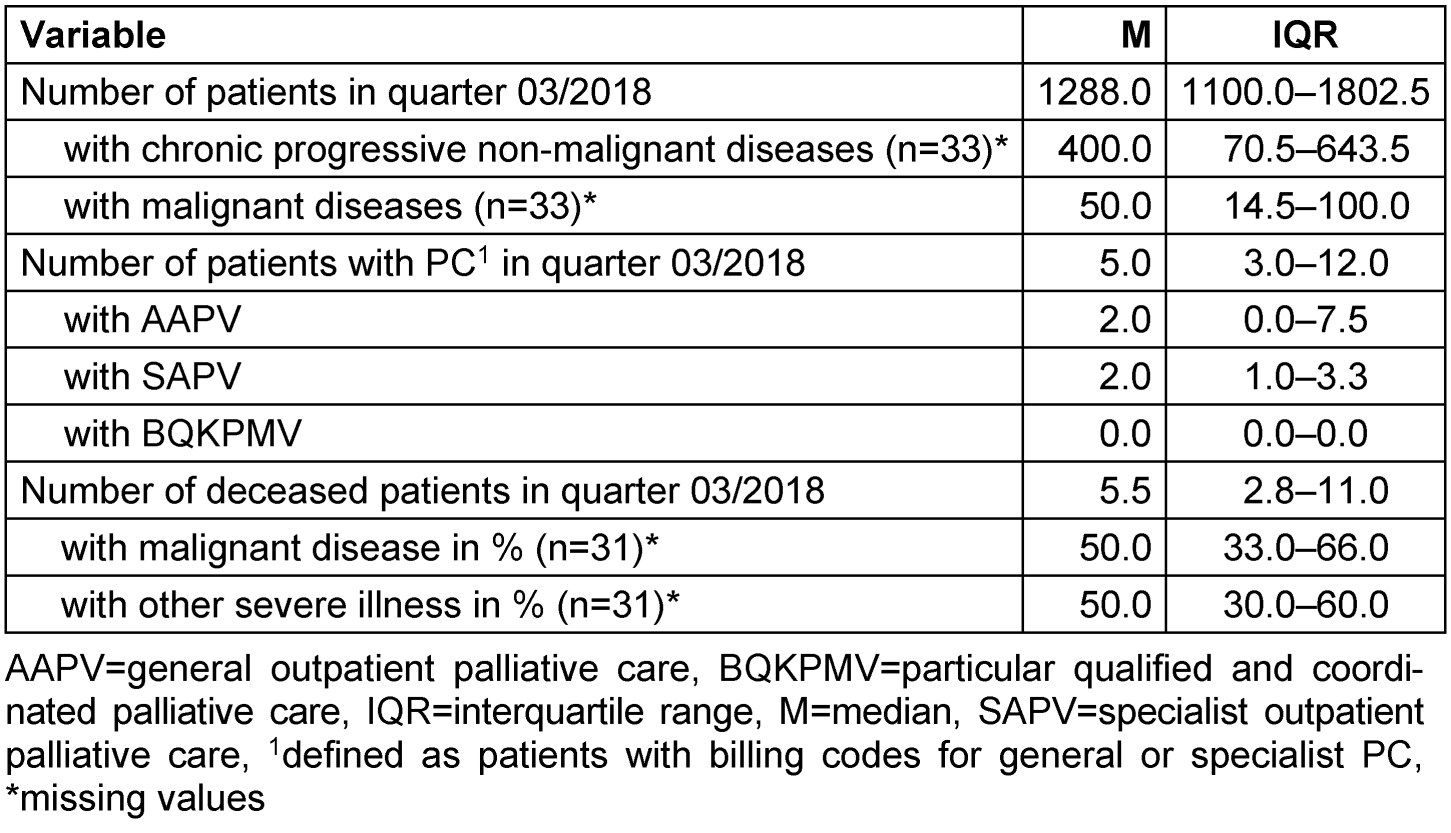
Characteristics of the patient population in the general practices (n=34)

**Table 4:**
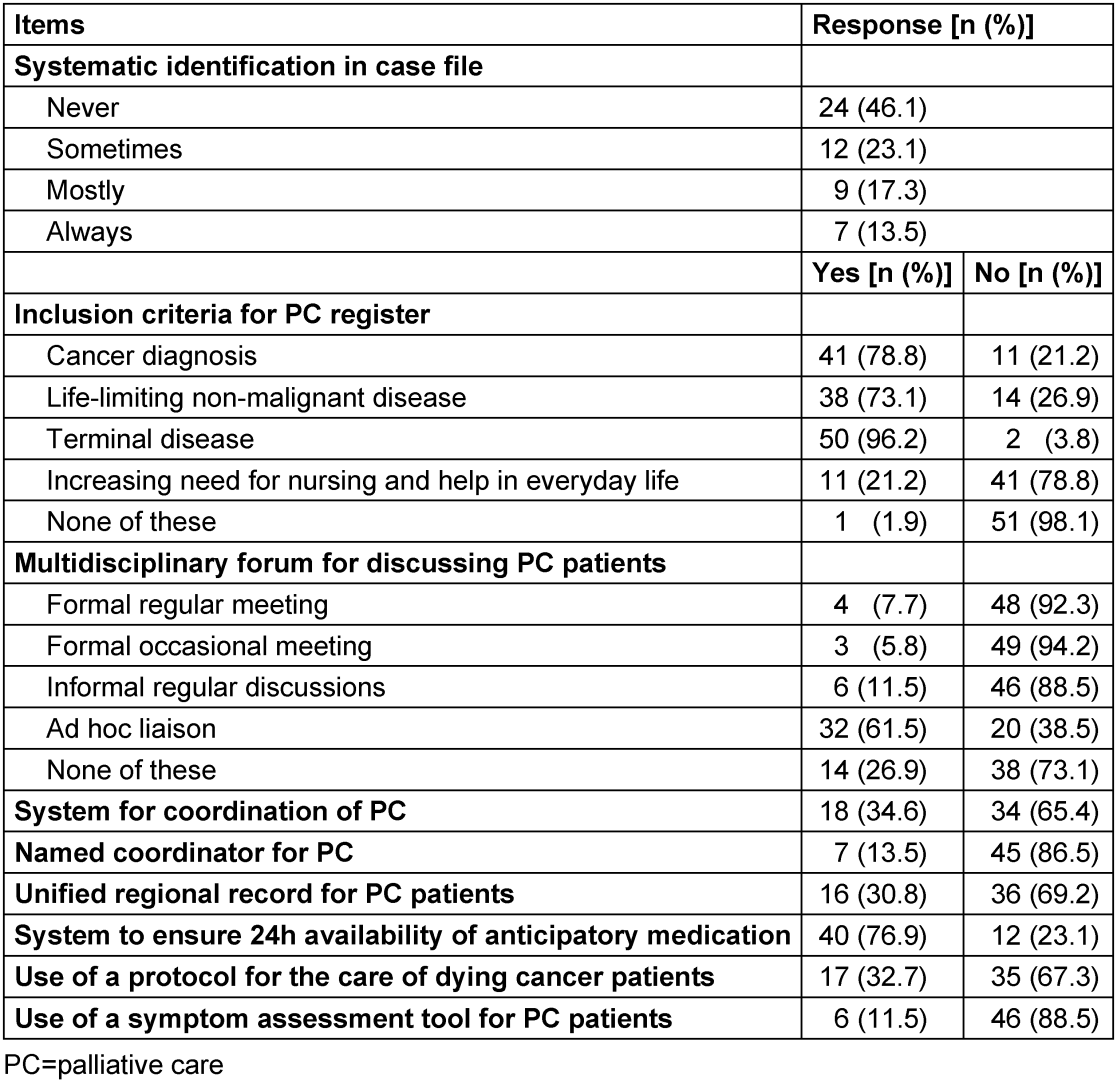
Subscale *practice organisation*

**Table 5:**
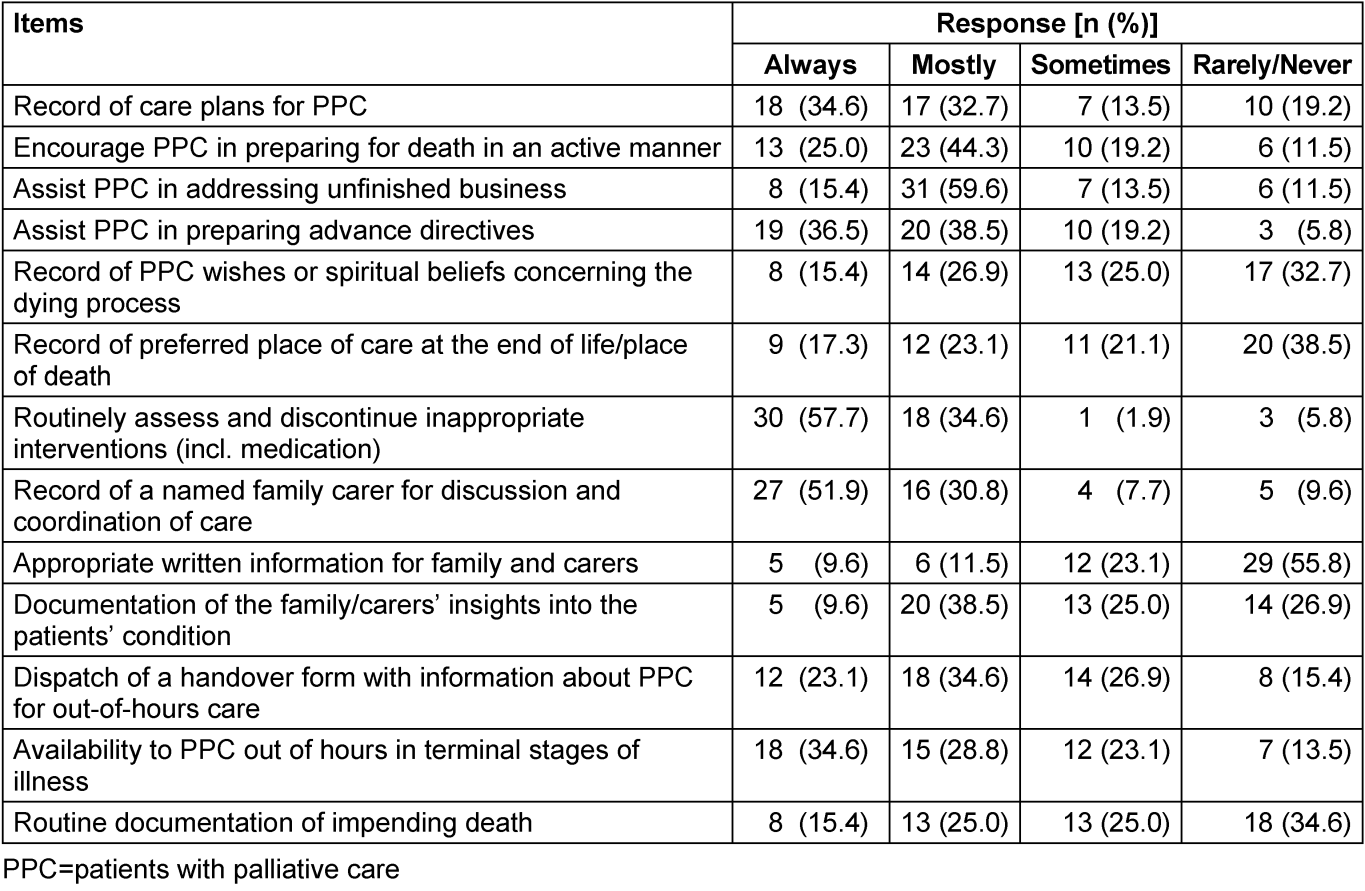
Subscale *clinical care*

**Table 6:**
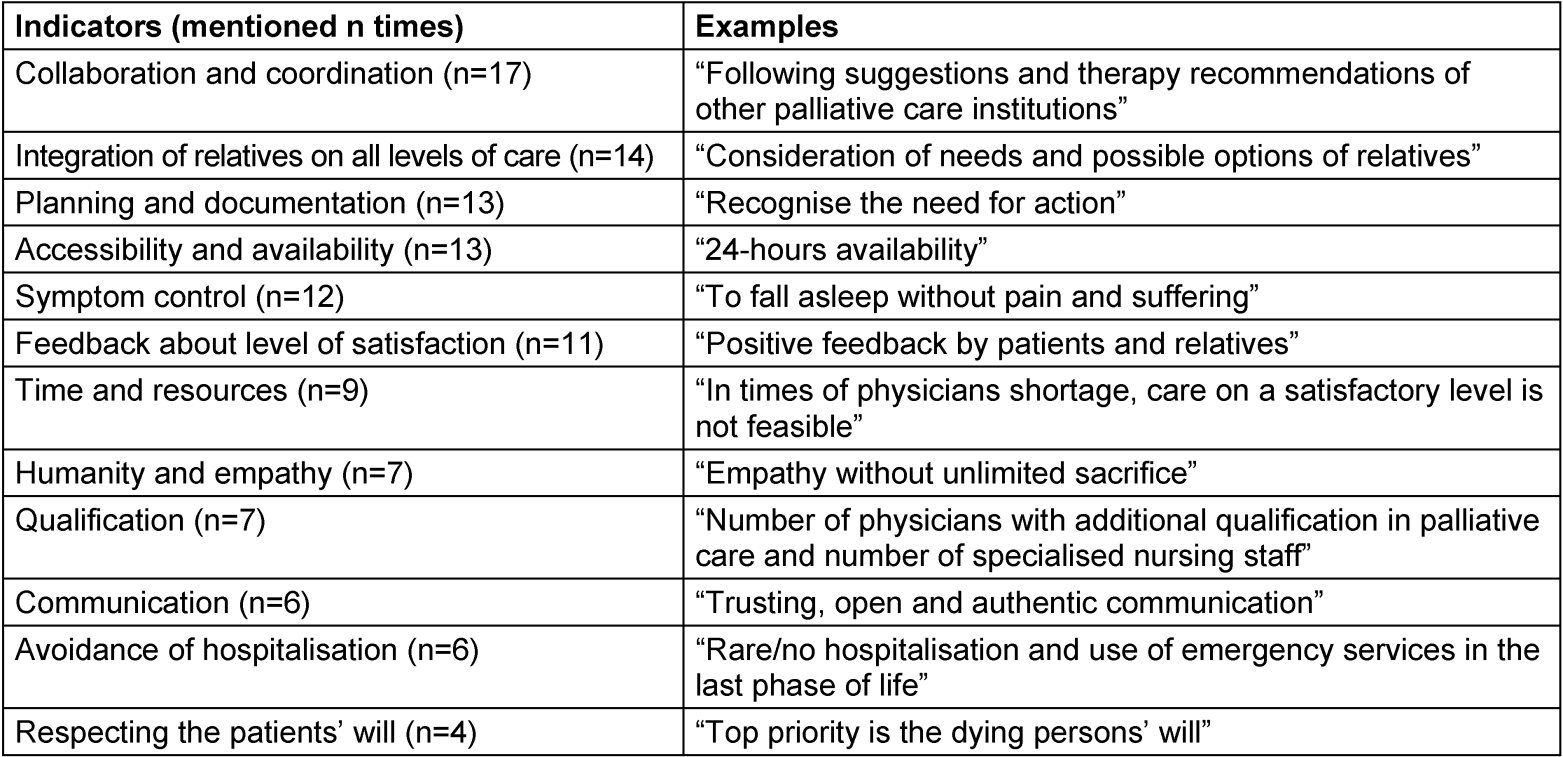
Categories and examples for indicators of good PC

**Table 7:**
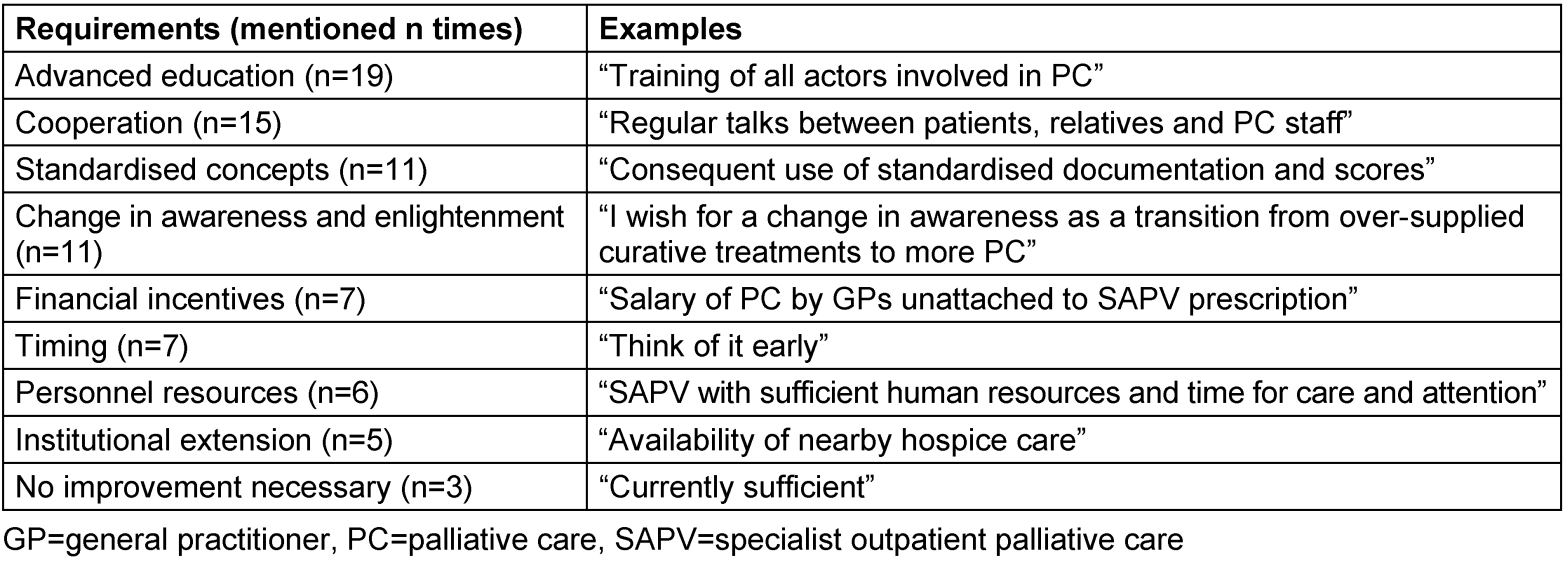
Categories and examples for requirements of an improvement of PC
